# The antenna of horse stomach bot flies: morphology and phylogenetic implications (Oestridae, Gasterophilinae: *Gasterophilus* Leach)

**DOI:** 10.1038/srep34409

**Published:** 2016-10-05

**Authors:** Dong Zhang, Xinyu Li, Xianhui Liu, Qike Wang, Thomas Pape

**Affiliations:** 1 Key Laboratory of Non-Invasive Research Technology for Endangered Species, School of Nature Conservation, Beijing Forestry University, Beijing 10083, China; 2 Department of Zoology, School of Nature Conservation, Beijing Forestry University, 35 Qinghua East Road, Beijing 100083, China; 3 University of California, Davis, One Shields Avenue, Davis, CA 95616, America; 4 School of Biosciences, University of Melbourne, Parkville, Melbourne, VIC 3010, Australia; 5 Natural History Museum of Denmark, University of Copenhagen, Copenhagen, Denmark

## Abstract

Antennae are among the most elaborate sensory organs in adult flies, and they provide rich information for phylogenic studies. The antennae of five out of eight species of *Gasterophilus* Leach (*G. haemorrhoidalis* (Linnaeus), *G. intestinalis* (De Geer), *G. nasalis* (Linnaeus), *G. nigricornis* (Loew) and *G. pecorum* (Fabricius)), were examined using scanning electron microscopy. The general morphology, including distribution, type, size, and ultrastructure of antennal sensilla were presented, and the definition of auriculate sensilla and sensory pits were updated and clarified. Eighteen antennal characters were selected to construct the first species-level phylogeny of this genus. The monophyly of *Gasterophilus* was supported by the presence of coeloconic sensilla III on the antennal arista. The species-level cladogram showed *G. pecorum* branching off at the base, and the remaining species forming the topology (*G. intestinalis*+ (*G. haemorrhoidalis*+ (*G. nasalis*+ *G. nigricornis*))). Our research shows the importance of the antennal ultrastructure as a reliable source for phylogenetic analysis.

Morphology is an indispensable and near-unlimited source for the study of dipteran systematics and evolution. While molecular techniques have gained much popularity in phylogenetic analyses, morphologists continue to apply novel morphological characters to reconstruct phylogenetic relationships of Diptera^1^,^2^,^3^. This is especially evident for small and complex structures that are difficult to investigate by traditional methods such as light microscopy^4^. Morphology undoubtedly has the potential for providing a large amount of information for systematics from the numerous character systems that still remain unexplored^5^.

Our morphological investigation focusses on the antennae, one of the most elaborate and morphologically diverse sensory organs. Antennae perform crucial functions in the life-cycle of most Diptera^6^,^7^,^8^,^9^,^10^,^11^,^12^,^13^,^14^,^15^,^16^, with their sensillar equipment fine-tuned by strong selection^17^. Diptera antennae offer an abundance of structures that provide excellent taxonomic and phylogenetic characters. Antennal ultrastructure has been used in classification and phylogenetic reconstruction at least since the beginning of last century^18^, yet new information continues to emerge^19^,^20^,^21^. There are numerous studies of antennal ultrastructure in Calyptratae, but they mainly focus on morphology, physiology and function speculation. The antennae of calyptrate flies offer a wealth of information about their evolutionary history, as well as insights into the ecology of each species, which has a valuable potential in phylogenetic studies^16^,^21^.

*Gasterophilus* Leach (horse stomach bot flies) is a relatively small genus that contains eight species worldwide^22^,^23^. These species are obligate intestinal parasites of equids (including horses, donkeys and zebras), sharing similar behaviour and life-cycles^23^,^24^,^25^. The unique morphology, biology and epidemiology of these flies have been documented extensively^23^,^26^,^27^,^28^,^29^,^30^,^31^.

In this study, we conducted a comprehensive morphological comparison of five species of *Gasterophilus*, including the distribution, types, size and ultrastructure of antennal sensilla. We used these data to construct the first (partial) species-level phylogeny for *Gasterophilus*.

## Materials and Methods

### Morphological study

Adult male and female specimens of *G. haemorrhoidalis* (Linnaeus), *G. intestinalis* (De Geer), *G. nasalis* (Linnaeus), *G. nigricornis* (Loew) and *G. pecorum* (Fabricius) were captured during 2009–2011 in Kalamaili Ungulate Nature Reserve, Xinjiang, northwest China. The specimens were pinned and air-dried on site before being deposited in Beijing Forestry University. Material of the Palaearctic species *G. inermis* (Brauer), as well as of the two Afrotropical species *G. meridionalis* (Pillers & Evans) and *G. ternicinctus* Gedoelst, were not available for the semi-destructive sampling necessary for the study of antennal sensilla. For general morphology, antennae were studied using an Olympus SZX16 stereoscopic microscope. Series of photographs were taken using a Canon 500D digital camera (Canon, Inc., Tokyo, Japan) mounted on the stereoscopic microscope and superimposed using Helicon Focus (Helicon Soft Ltd, Kharkov, Ukraine) on a Windows 7 platform. All micrographs were processed in Adobe Photoshop CS5 (Adobe Systems, Inc., San Jose, CA, USA).

To prepare samples for scanning electron microscopy (SEM) documentation, the heads of exemplar specimens were excised and rinsed in PBS buffer (pH 7.0) to remove surface debris. For further cleaning, the antennal funiculus (including arista) was dissected from the remaining antenna, cleaned with detergent solution in an ultrasonic cleaner (50 °C, 15 min) and dehydrated in a graded ethanol series. The prepared samples were air-dried and mounted on aluminium stubs with double-sided adhesive tape and coated with gold before observing in a HITACHI S34Q scanning electron microscope (Hitachi Corp., Tokyo, Japan) at the Microscopy Core Facility, Biological Technology Center, Beijing Forestry University (Beijing, China). The length, basal diameter, distal dilation diameter, and distribution of antennal sensilla were measured.

As shown by Zhang *et al*.^12^, the types of antennal sensilla in male *G. nigricornis* are highly similar to those of the female, so for this species only the distribution of sensilla on the female antenna is given in this paper. The terminology follows Zhang *et al*.^12^,^13^,^14^,^15^,^16^.

### Phylogenetic analysis

Eighteen characters (13 binary, 5 multistate) were included in the analysis, which covered characters from antennal scape, pedicel, funiculus (including arista) and sensilla (Appendix 1). The matrix (Appendix 2) was compiled using MESQUITE 1.05^32^. No data are available on the antennal ultrastructure for the genus *Gyrostigma* Brauer, which is the most probable sister-group to *Gasterophilus*^33^, and no specimens are available for the semi-destructive sampling required for proper SEM-documentation. We chose *Hypoderma lineatum* (Villers) (Hypodermatinae) and *Rhinoestrus purpureus* (Brauer) (Oestrinae) as outgroup representatives, as the antennal ultrastructure of these two species has been extensively documented by Liu *et al*.^9^ and Li *et al*.^10^, respectively. For proper rooting of the cladogram, we used *Lucilia sericata* (Meigen) (Calliphoridae) as a more distant outgroup whose Antennal ultramorphology was reported by Zhang *et al*.^15^.

Phylogenetic analyses were performed under the parsimony criterion with the program TNT 1.1^34^. All multistate characters were considered to have their states forming linear transformation series as indicated under the relevant character following the description of character states. They were treated as either additive (=ordered) or non-additive as given in the discussion. Exhaustive searches using implicit enumeration were conducted under equal weights (=unweighted) and with implied weighting^35^ under a range of k-values as given in the discussion. Branches were collapsed if the maximum branch length was zero.

Branch support for individual nodes was estimated by Bremer’s decay index^35^,^36^. Suboptimal trees allowing for five extra steps were produced in TNT to calculate absolute Bremer support values.

## Results and Discussion

### General description of the antennal morphology in *G. haemorrhoidalis, G. intestinalis, G. nasalis, G. nigricornis* and *G. pecorum*

**Figure 1:**
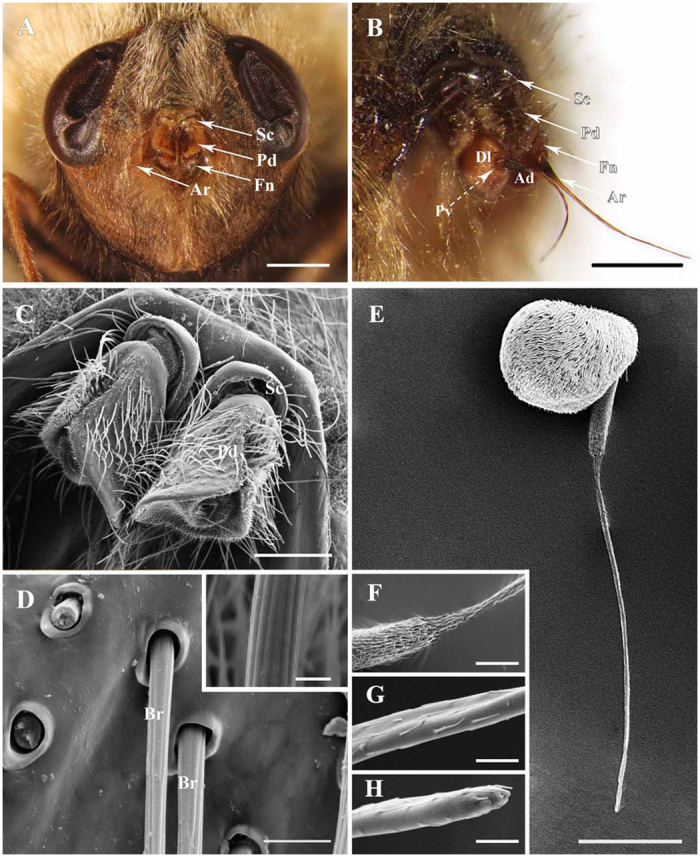
Features on the head of adult *Gasterophilus pecorum*. (**A**) Antennae located centrally between compound eyes. (**B**) Anterodorsal surface, dorsolateral margin and posteroventral surface of antenna in resting position. (**C**) Antennal scape and pedicel. (**D**) Base and (inset) middle parts of setae on antennal pedicel. (**E**) Flagellum. (**F**–**H**) Base, middle and tip of arista. Scale bars: A = 1 mm, B = 500 μm, C = 200 μm, D = 100 μm, 5 μm in inset, E = 250 μm, F = 50 μm, G, H = 10 μm. Abbrevations: Ad, anterodorsal surface; Ar, arista; Br, bristle; Dl, dorsolateral margin; Fn, funiculus; Pd, pedicel; Pv, posteroventral surface; Sc, scape.

**Figure 2:**
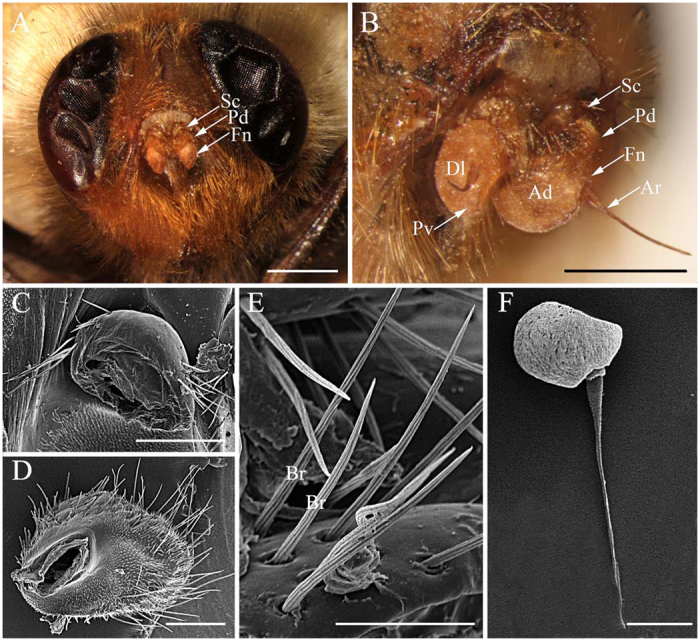
Features on the head of adult *Gasterophilus nasalis*. (**A**) Antennae located centrally between compound eyes. (**B**) Antenna in resting position, showing anterodorsal surface, dorsolateral margin in front and posteroventral surface. (**C**) Antennal scape. (**D**) Antennal pedicel. (**E**) Bristle (mechanoreceptor) on antennal pedicel. (**F**) Flagellum. Scale bars: A = 1 mm, B, F = 250 μm, C = 200 μm, D = 150 μm, E = 50 μm. Abbrevations: Ad, anterodorsal surface; Ar, arista; Br, bristle; Dl, dorsolateral margin; Fn, funiculus; Pd, pedicel; Pv, posteroventral surface; Sc, scape.

**Figure 3:**
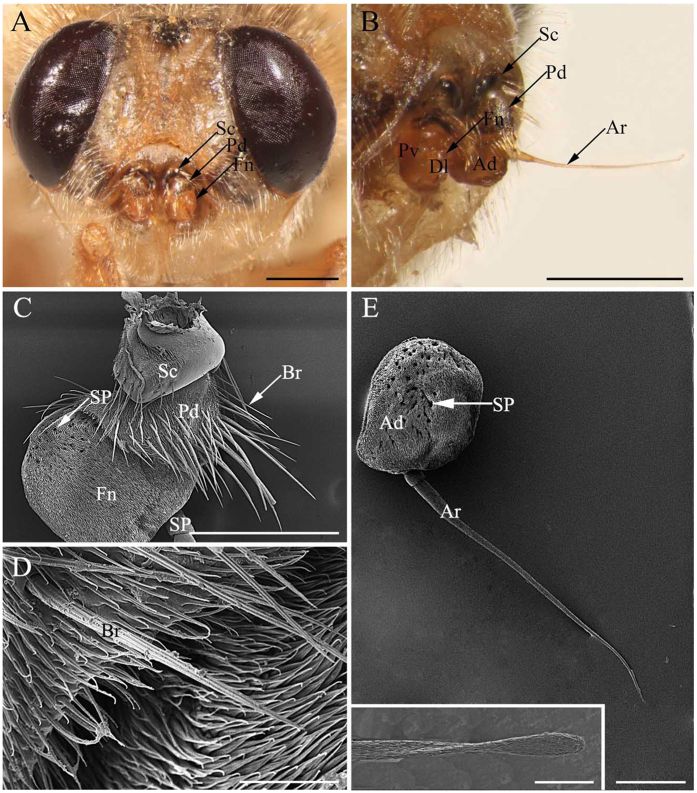
Features on the head of adult *Gasterophilus intestinalis.* (**A**) Antennae located centrally between compound eyes. (**B**) Antenna in resting position, showing anterodorsal surface, dorsolateral margin in front and posteroventral surface. (**C**) Antennal scape and pedicel. (**D**) Bristle (mechanoreceptor) on antennal pedicel. (**E**) Anterodorsal surface of antennal funiculus and (inset) aristal tip. Scale bars: A, B = 1mm, C = 200 μm, D = 25 μm, E = 250 μm, 50 μm in inset. Abbrevations: Ad, anterodorsal surface; Ar, arista; Br, bristle; Dl, dorsolateral margin; Fn, funiculus; Pd, pedicel; Pv, posteroventral surface; Sc, scape. SP, sensory pit.

**Figure 4:**
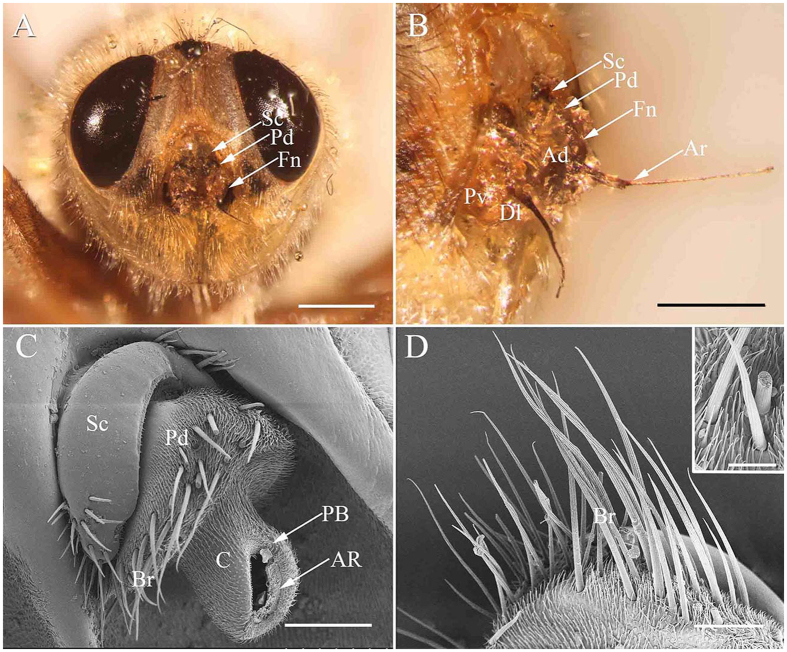
Features on the head of adult *Gasterophilus haemorrhoidalis.* (**A**) Antennae located centrally between compound eyes. (**B**) Antenna in resting position, showing anterodorsal surface, dorsolateral margin in front and posteroventral surface. (**C**) Antennal scape and pedicel. (**D**) Bristle (mechanoreceptor) on antennal pedicel. Scale bars: A = 1 mm, B = 0.5 mm, C = 100 μm, D = 50 μm, 20 μm in inset. Abbrevations: Ad, anterodorsal surface; AR, annular ridge; Ar, arista; Br, bristle; C, cone; Dl, dorsolateral margin; Fn, funiculus; PB, pedicellar button; Pd, pedicel; Pv, posteroventral surface; Sc, scape.

Like other schizophoran Diptera, the antennae of these five species are a pair of segmented sensory appendages arising from membranous sockets between the compound eyes and below the lunule (Figs 1A–C, 2A–C, 3A,B and 4A–C). Each antenna consists of a proximal scape (Sc), a pedicel (Pd) and a distal flagellum composed of a pyriform funiculus (Fn) and a slender, bristle-like arista (Ar) (Figs 1A–C,E, 2A–D,F, 3A–C,E and 4A–C).

**Figure 5:**
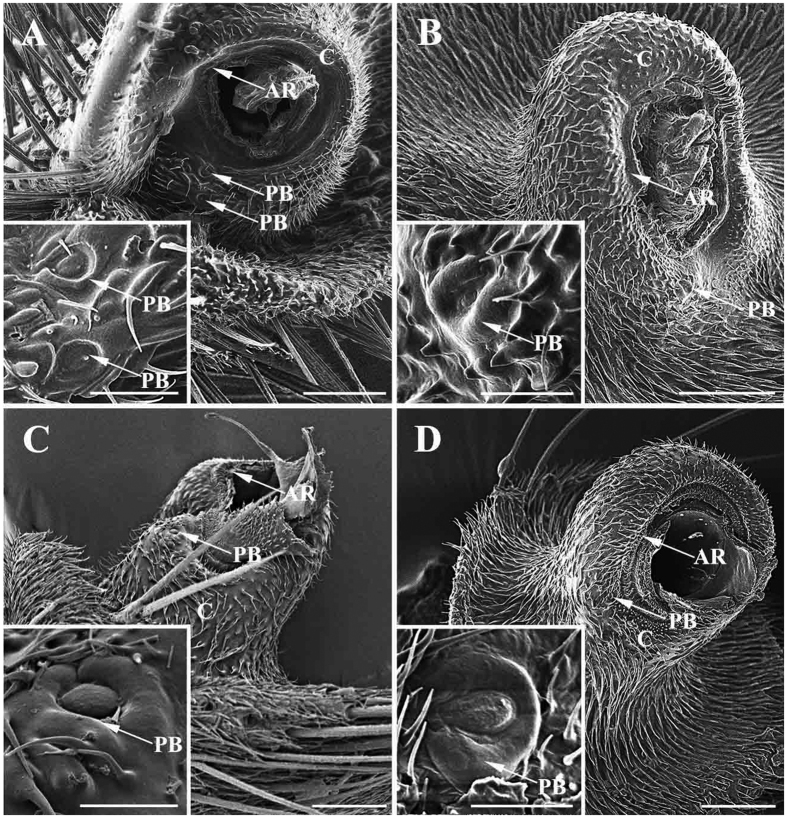
Details of antennal pedicel after removal of antennal funiculus and (inset) magnification of pedicellar button. (**A**) *Gasterophilus pecorum*. (**B**) *G. nasalis*. (**C**) *G. intestinalis*. (**D**) *G. haemorrhoidalis*. Scale bars: A = 50 μm, 15 μm in inset, B, C = 50 μm, 10 μm in inset, D = 50 μm, 5 μm in inset. Abbreviations: PB, pedicellar button; AR, annular ridge C, cone.

The scape, the first and shortest segment of the antenna, has a smooth cuticular surface without microtrichia. The pedicel (Figs 1A–C, 2A–C, 3A–C and 4A–C), the second segment, is flattened and disc-like and is covered with microtrichia on the surface. In *G. pecorum*, the pedicel is elongated and is the longest segment (Fig. 1A–C). Mechanoreceptors (Figs 1C,D, 2C–E, 3C,D and 4C,D) are distributed both on the scape and pedicel. These are short, straight setae or bristles of varying length and with the usual longitudinal grooves on the cuticular surface. The pedicellar button (PB) (Fig. 5) is a button-like structure situated on the articular surface near the pedicellar cleft, and it consists of a circular dome centrally and a slightly convex (*G. intestinalis* and *G. pecorum*) or distinctly ridgy (*G. haemorrhoidalis*, *G. nasalis*, *G. nigricornis*) ring peripherally (Fig. 5). *Gasterophilus pecorum* has two buttons on the pedicel (Fig. 5A), whereas there is only one in the other four species.

**Figure 6:**
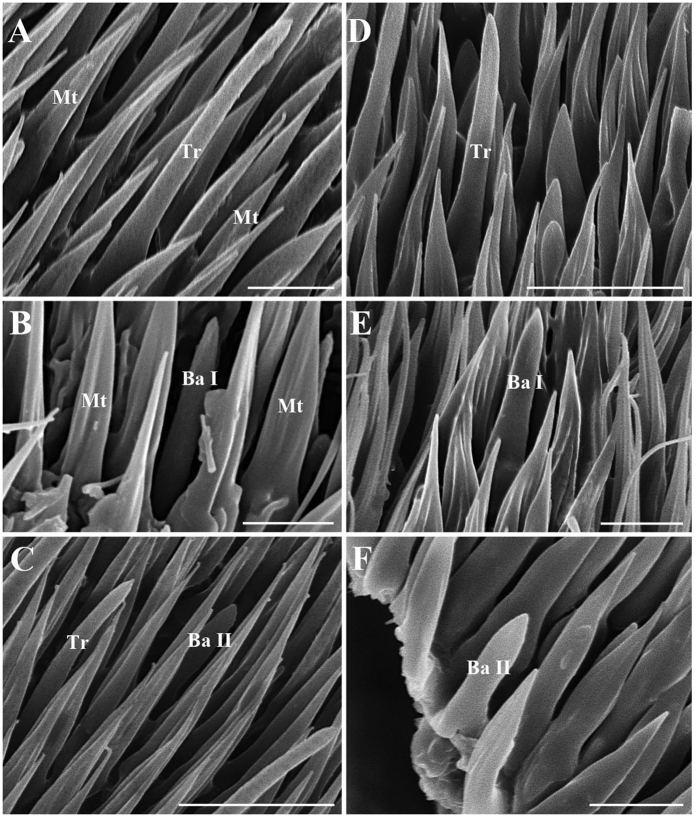
Scanning electron micrographs of trichoid sensilla and basiconic sensilla on antennal funiculus in *Gasterophilus pecorum* and *G. nasalis*. (**A**) Trichoid sensilla in *G. pecorum*. B Basiconic sensilla I in *G. pecorum*. C Basiconic sensilla II in *G. pecorum*. D Trichoid sensilla in *G. nasalis*. E Basiconic sensilla I in *G. nasalis*. F Basiconic sensilla II in *G. nasalis*. Scale bars: A, B, E, F = 5 μm, C, D = 10 μm. Abbreviations: Ba, basiconic sensilla; Ba I, basiconic sensilla I; Ba II, basiconic sensilla II; Mt, microtrichia; Tr, trichoid sensilla.

**Figure 7:**
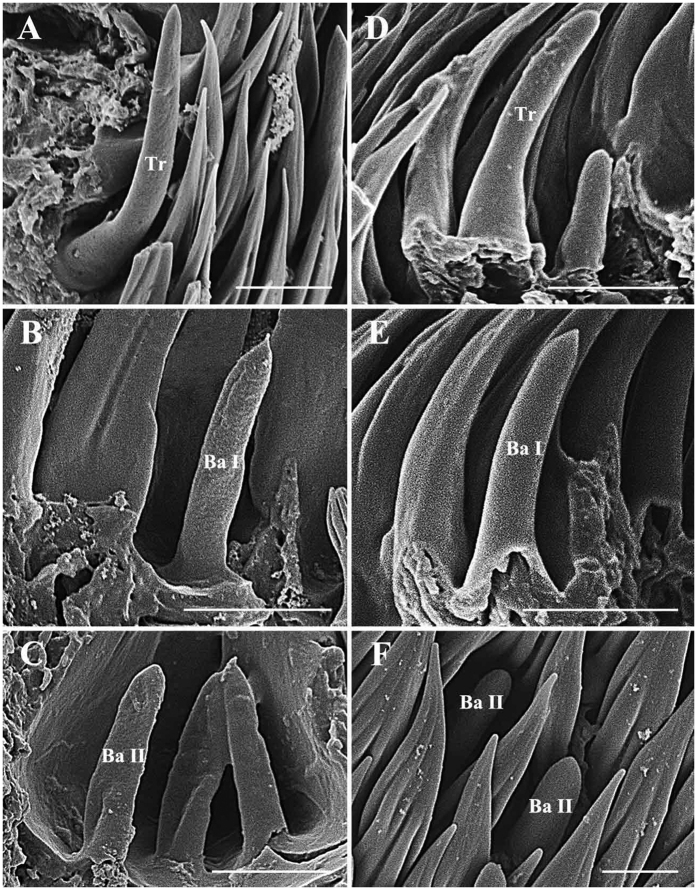
Scanning electron micrographs of trichoid sensilla and basiconic sensilla on antennal funiculus in *Gasterophilus intestinalis* and *G. haemorrhoidalis*. (**A**) Trichoid sensilla in *G. intestinalis*. (**B**) Basiconic sensilla I in *G. intestinalis*. (**C**) Basiconic sensilla II in a sensory pit in *G. intestinalis*. (**D**) Trichoid sensilla in *G. haemorrhoidalis*. E Basiconic sensilla I in *G. haemorrhoidalis*. F Basiconic sensilla II in *G. haemorrhoidalis*. Scale bars: A, B, C, D, E = 5 μm, F = 2.5 μm. Abbreviations: Ba, basiconic sensilla; Ba I, basiconic sensilla I; Ba II, basiconic sensilla II; Tr, trichoid sensilla.

**Figure 8:**
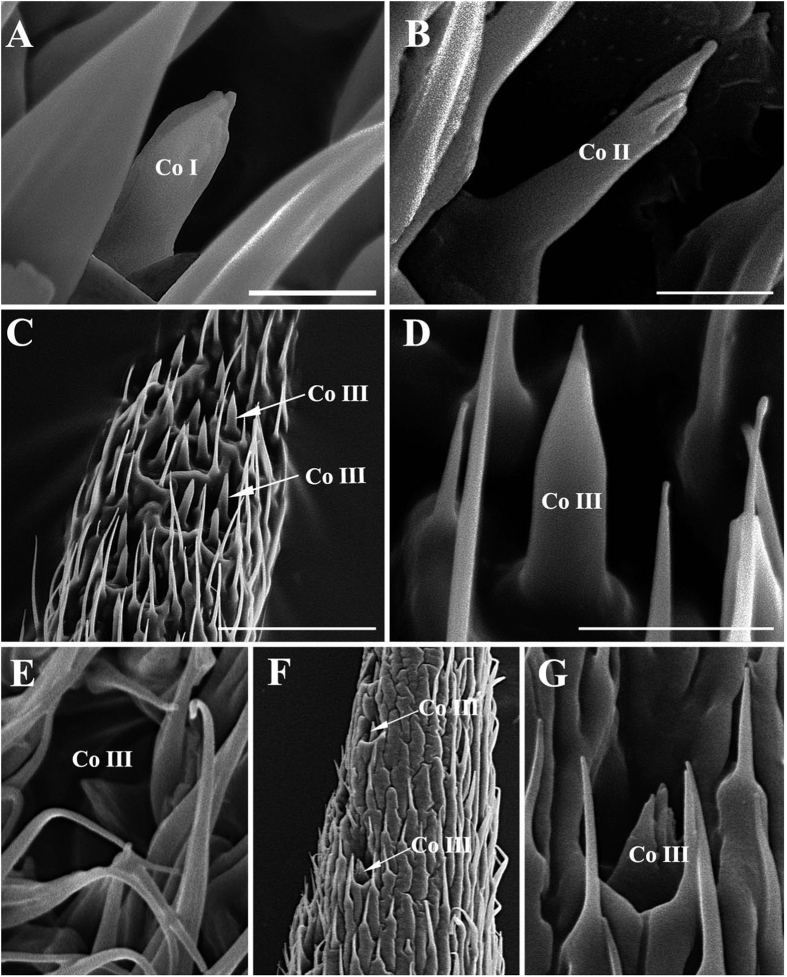
Scanning electron micrographs of coeloconic sensilla on antennal funiculus and arista in *Gasterophilus pecorum* and *G. nasalis*. (A) Coeloconic sensilla I in *G. pecorum*. (**B**) Coeloconic sensilla II in *G. pecorum*. (**C**) Coeloconic sensilla III on basal part of arista in *G. pecorum*. (**D**) Magnification of coeloconic sensilla III in C. (**E**) Coeloconic sensilla III in depression on antennal funiculus in *G. nasalis*. (**F**) Coeloconic sensilla III on basal part of arista in *G. nasalis*. (**G**) Coeloconic sensilla III in F. Scale bars: A, B, E = 2.5 μm, C = 30 μm, D, G = 5 μm, F = 25 μm. Abbreviations: Co I, coeloconic sensilla I; Co II, coeloconic sensilla II; Co III, coeloconic sensilla III.

**Figure 9:**
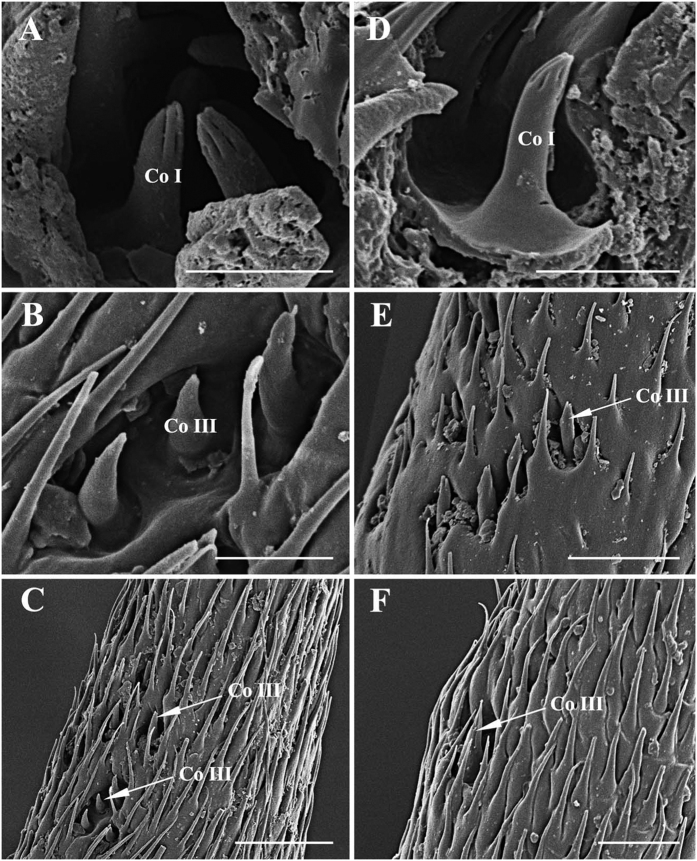
Scanning electron micrographs of coeloconic sensilla on antennal funiculus and arista in *Gasterophilus intestinalis* and *G. haemorrhoidalis*. (**A**) Coeloconic sensilla I within a sensory pit in *G. intestinalis.* (**B**) Coeloconic sensilla III on antennal arista in *G. intestinalis.* (**C**) Coeloconic sensilla III on basal part of arista in *G. intestinalis*. (**D**) An coeloconic sensillum I in *G. haemorrhoidalis*. (**E**) Coelocronic sensilla III on antennal arista in *G. haemorrhoidalis.* (**F**) Coeloconic sensilla III on basal part of arista in *G. haemorrhoidalis.* Scale bars: A, B, D = 5 μm, C = 20 μm, E, F = 10 μm. Abbreviations: Co I, coeloconic sensilla I; Co III, coeloconic sensilla III.

**Figure 10:**
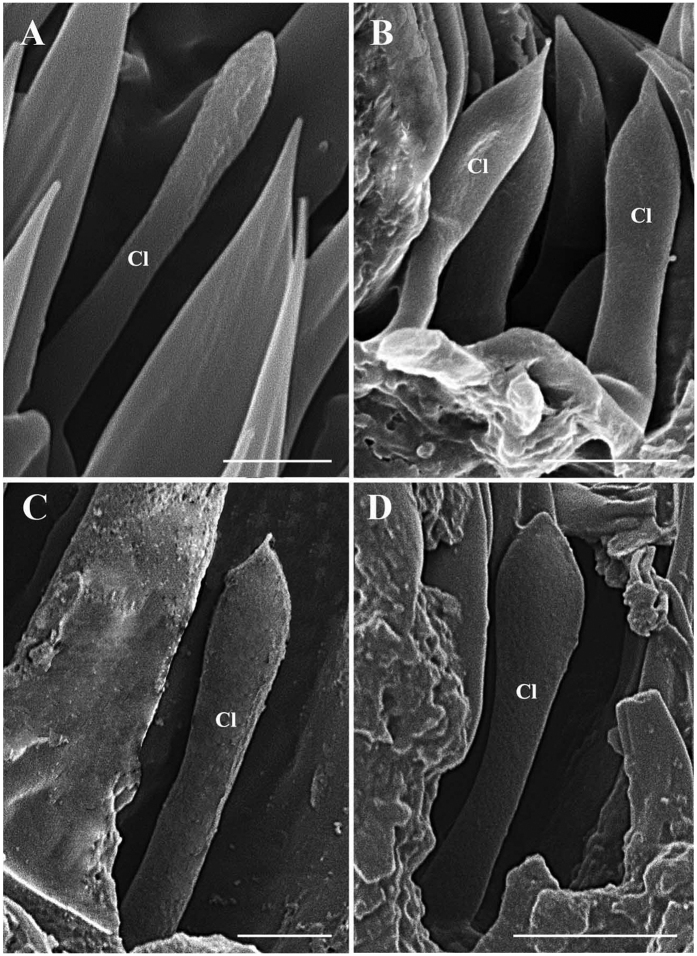
Scanning electron micrographs of clavate sensilla on antennal funiculus in *Gasterophilus nasalis* and *G. intestinalis*. (**A**) Clavate sensillum in shallow depression in *G. nasalis*. (**B**) Clavate sensilla within a sensory pit in *G. nasalis*. (**C**) Clavate sensillum in shallow depression in *G. intestinalis*. (**D**) Clavate sensillum in shallow depression in *G. haemorrhoidalis*. Scale bars: A, C = 2.5 μm, B, D = 5 μm. Abbreviation: Cl, clavate sensilla.

**Figure 11:**
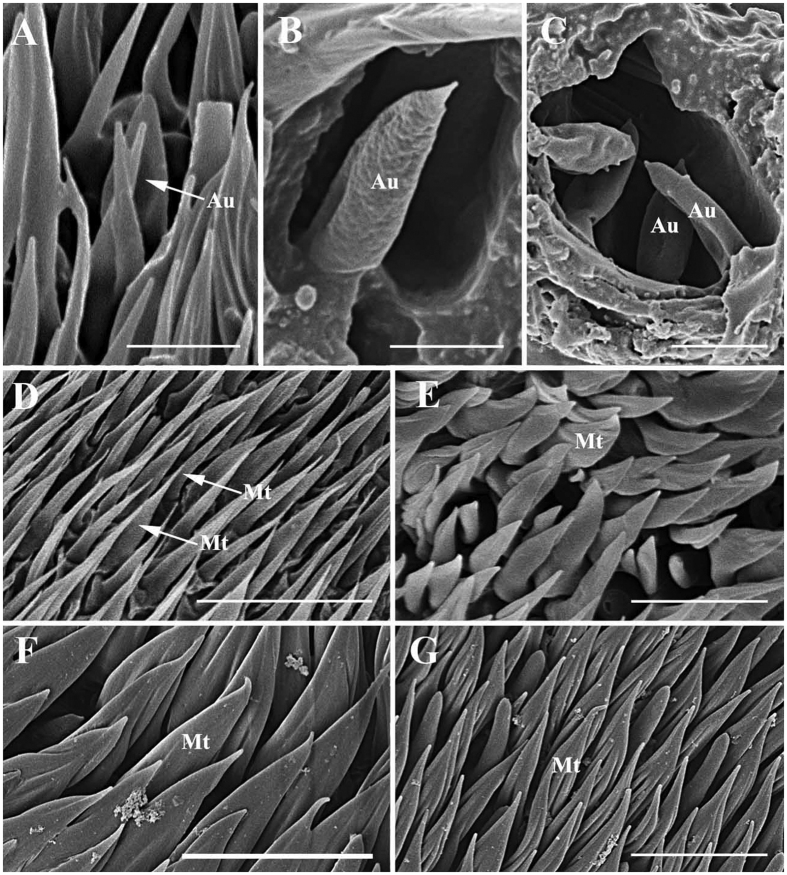
Scanning electron micrographs of auriculate sensilla on antennal funiculus and microtrichiae. (**A**) Auriculate sensillum in *Gasterophilus nasalis*. (**B**) Auriculate sensillum in *G. intestinalis*. (**C**) Auriculate sensilla clustered in a sensory pit in *G. haemorrhoidalis*. (**D**) Microtrichiae on antennal funiculus in *G. pecorum*. (**E**) Microtrichiae on antennal funiculus in *G. nasalis*. (**F**) Microtrichiae on antennal funiculus in *G. intestinalis*. (**G**) Microtrichiae on antennal funiculus in *G. haemorrhoidalis.* Scale bars: A, C = 5 μm, B = 2.5 μm, D = 20 μm, E, F, G = 10 μm, F = 10 μm, G = 10 μm. Abbreviations: Au, auriculate sensilla; Mt, microtrichia.

**Figure 12:**
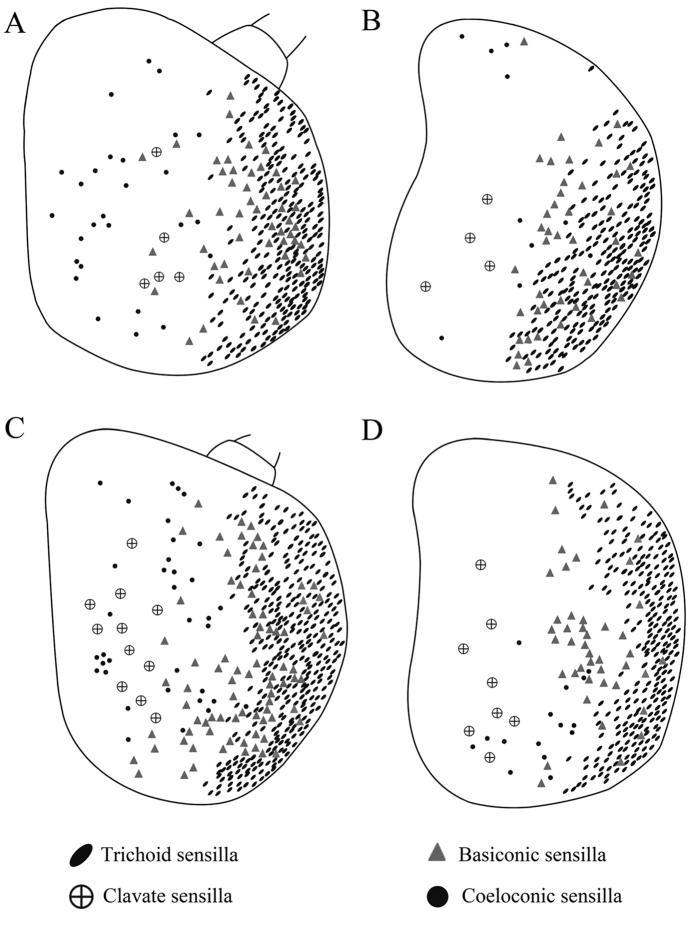
Distribution of four types of sensilla on anterodorsal surface and dorsolateral margin (**A** male, **C** female) and posteroventral surface (**B** male, **D** female) of antennal funiculus in *Gasterophilus pecorum*.

**Figure 13:**
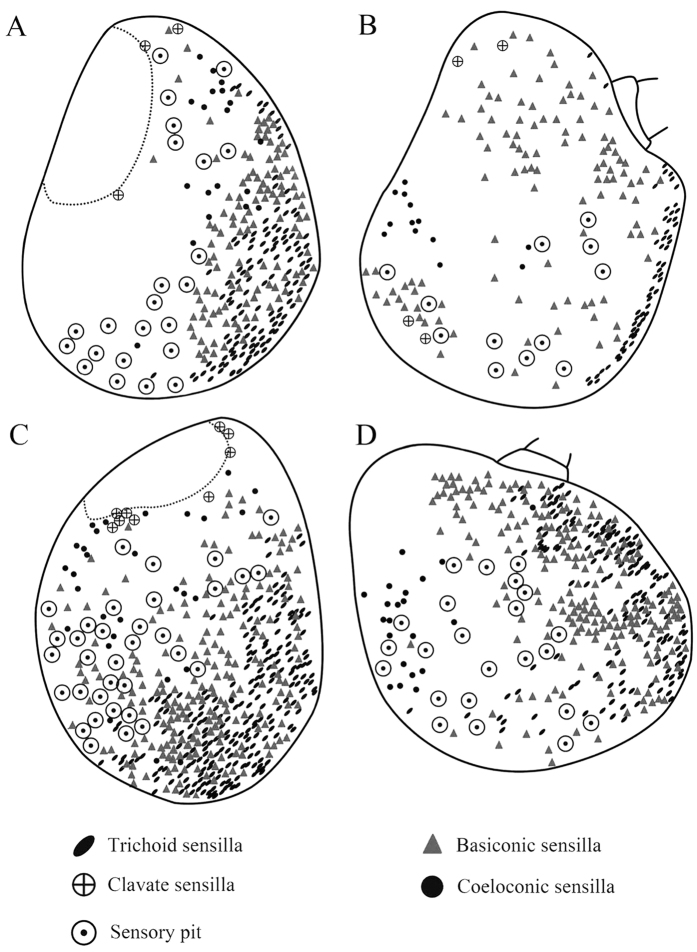
Distribution of four types of sensilla on anterodorsal surface and dorsolateral margin (**A** male, **C** female) and posteroventral surface (**B** male, **D** female) of antennal funiculus in *Gasterophilus nasalis*.

**Figure 14:**
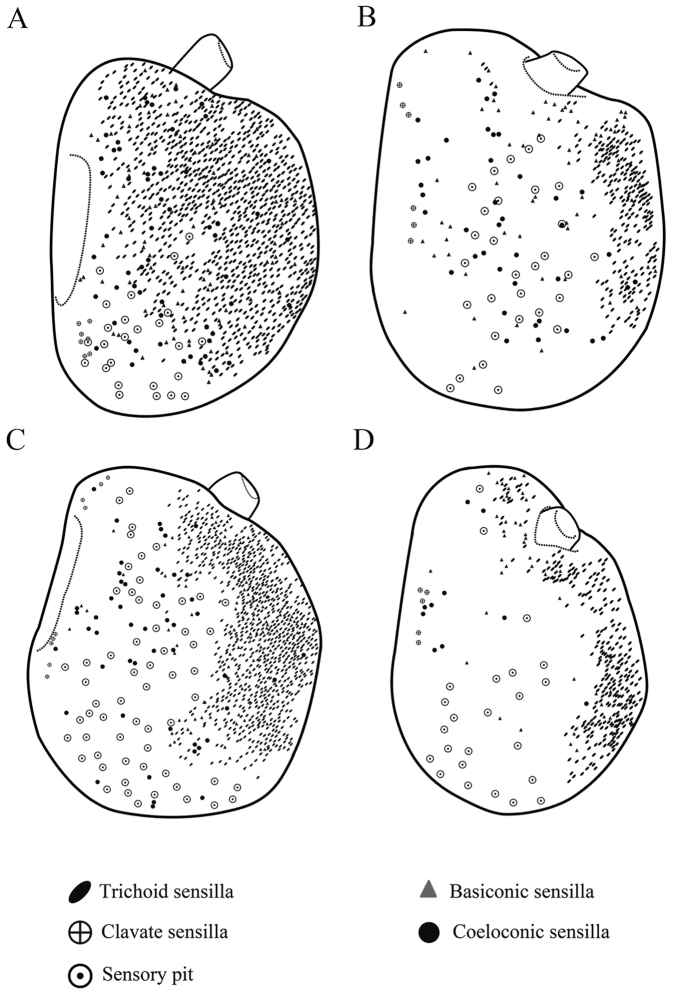
Distribution of four types of sensilla on anterodorsal surface and dorsolateral margin (**A** male, **C** female) and posteroventral surface (**B** male, **D** female) of the antennal funiculus in *Gasterophilus intestinalis*.

**Figure 15:**
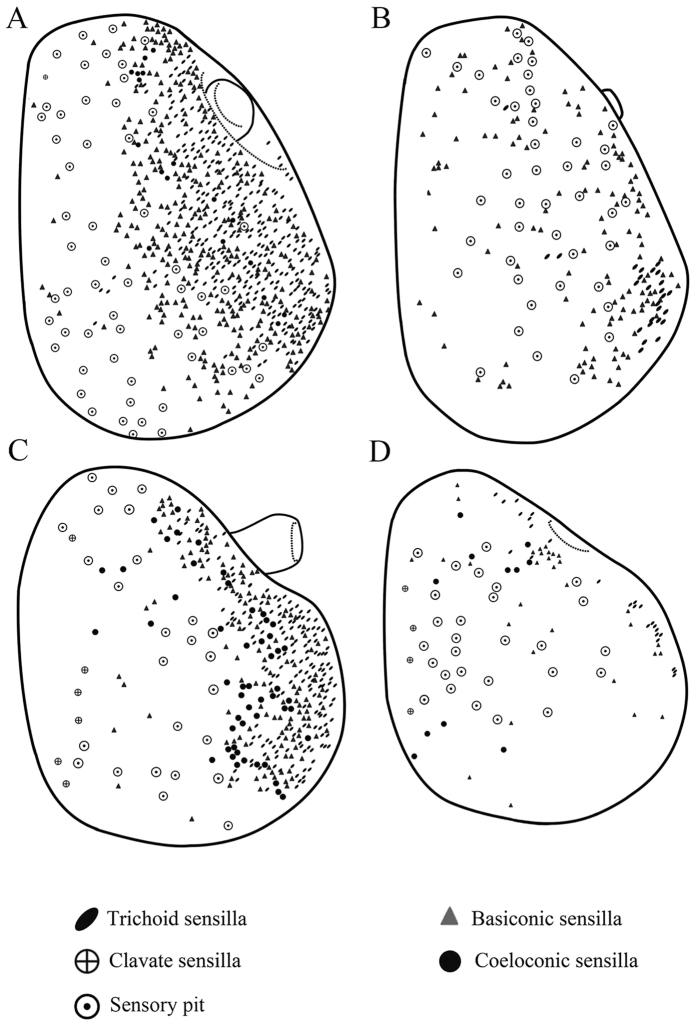
Distribution of four types of sensilla on anterodorsal surface and dorsolateral margin (**A** male, **C** female) and posteroventral surface (**B** male, **D** female) of antennal funiculus in *Gasterophilus haemorrhoidalis.*

**Figure 16:**
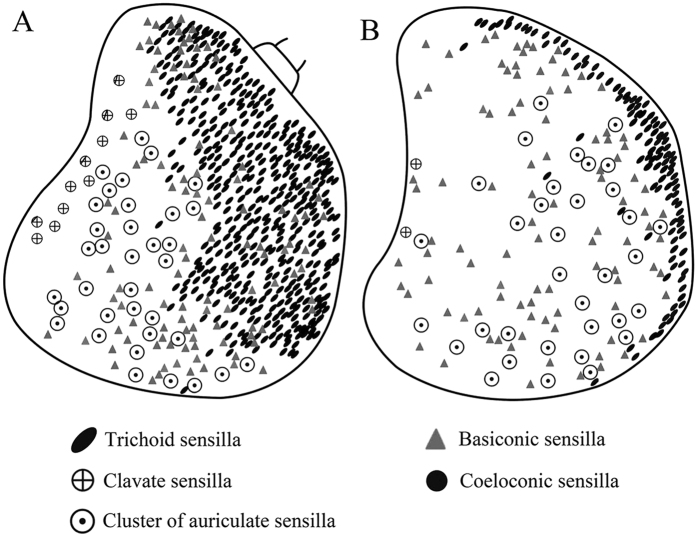
Distribution of four types of sensilla on anterodorsal surface and dorsolateral margin (**A**) and posteroventral surface (**B**) of antennal funiculus in female *Gasterophilus nigricornis*.

The funiculus is the most conspicuous segment of the antenna, housing a variety of sensilla. It is roughly pyriform and can be divided into three aspects: the anterodorsal surface (Ad), the dorsolateral surface (with the arista) (Dl), and the posteroventral surface (Pv) (Figs 1B, 2B, 3B and 4B). A total of five types of antennal sensilla are found, including trichoid sensilla (Figs 6A–D and 7A–D), basiconic sensilla (Figs 6B,C,E,F and 7B,C,E,F), coeloconic sensilla (Figs 8 and 9), clavate sensilla (Fig. 10) and auriculate sensilla (Fig. 11A–C). The length, basal diameter, and distal dilation diameter of these sensilla for both males and females are summarized in Tables 1, 2, 3 and 4, and their distribution is shown in Figs 12, 13, 14, 15 and 16. The arista consists of one or two short basal segments and one long distal segment that tapers gradually (Figs 1A,B,E–H, 2B,F, 3B,E and 4B). The short basal segment(s) are without sensilla, while the distal aristal segment is equipped with coeloconic sensilla III.

### General description of the funicular sensilla

#### Trichoid sensilla

Trichoid sensilla (Tr) are the longest sensilla on the antennal funiculus (Figs 6A–D and 7A–D; Tables 1, 2, 3 and 4). They are slender, tapering and blunt-tipped structures extending above the microtrichia at various lengths. The trichoid sensilla are concentrated on the anterodorsal surface and show a conspicuous density gradient with the number increasing from base to tip of the funiculus (Figs 12, 13, 14, 15 and 16).

#### Basiconic sensilla

Basiconic sensilla (Ba) are shorter than trichoid sensilla and less widespread on the funicular surface (Figs 6B,C,E,F, 7B,C,E,F, 12, 13, 14, 15 and 16, Tables 1, 2, 3 and 4). Two subtypes (Ba I, II) of basiconic sensilla can be distinguished by their size in each species. Ba I (Figs 6B,E and 7B,E) and Ba II (Figs 6C,F and 7C,F) are both digitiform with a cylindrical shaft, and have abruptly blunt tips, but Ba I is 1.04 (*G. intestinalis*) to 2.60 (*G. haemorrhoidalis*) times longer and 1.40 (*G. haemorrhoidalis*) to 1.71 (*G. nasalis*) times thicker at base than Ba II (Tables 1, 2, 3 and 4).

#### Coeloconic sensilla

Coeloconic sensilla (Co) are the shortest of the sensilla, and are located in sunken cavities (Figs 8 and 9; Tables 1, 2, 3 and 4). Three subtypes (Co I, II, III) of Co can be distinguished: Co I and Co II are distributed on the mid-proximal region of the anterodorsal and the posteroventral surface of the funiculus, whereas Co III is found only on the arista. Co I (Figs 8A,E and 9A,D) is a short peg with deep, longitudinal grooves over the distal 1/3–1/4 of the sensillum, and it is found in all five species studied. Co II (Fig. 8B) is a relatively long peg with an irregular pattern on the terminal part. This type is found only in *G. pecorum*. Co III is usually short and smooth (Figs 8C,D,F,G and 9B,C,E,F), except in *G. nasalis*, in which it is approximately triangular in shape and with grooves (Fig. 8E–G), and found exclusively at the base of the distal aristal segment. In *G. intestinalis* and *G. pecorum*, Co III are clustered in shallow depressions, while in *G. haemorrhoidalis*, *G. nasalis*, and *G. nigricornis*, they are distributed singly (Figs 8 and 9).

#### Clavate sensilla

The clavate sensilla (Cl) are characterised by a subapical dilation or swelling, giving them a club-like or spatulate appearance. This type of sensillum is seated in a superficial cavity (Fig. 10) and can only be observed on the most proximal region of the funiculus (Figs 12, 13, 14, 15 and 16). In *G. nasalis* (Fig. 10B) and *G. nigricornis*, each clavate sensillum has a short, tapering tip, while in the other species of *Gasterophilus*, the Cl is more abruptly tapered with the tip broadly rounded (Fig. 10A) or angulated (Fig. 10C,D). Additionally, clavate sensilla are distributed either singularly on the surface (Fig. 10A) or clustered in pits (Fig. 10B).

#### Auriculate sensilla

In *Gasterophilus* spp., the auriculate sensilla (Au) are gradually tapered from the base, with a length-width ratio <3 (Tables 1, 2, 3 and 4), somewhat resembling the ear of a rabbit (Fig. 11A–C). Au are distributed either singly on the surface or clustered in sensory pits.

Auriculate sensilla were first discovered by Setzu *et al*.^37^ in *Protophormia terraenovae* (Robineau-Desvoidy) (Calliphoridae) and described as ear- or spoon-like formations presenting a concavely indented or sunken surface distally. In this study, we observed this type of sensilla in four species (*G. haemorrhoidalis*, *G. intestinalis*, *G. nasalis* and *G. nigricornis*). It should be noted that distinguishing antennal sensilla solely by their cuticular surface concavity can be misleading, since several sensilla that we observed (e.g., clavate sensilla) had partly sunken cuticular surfaces. They were situated amongst morphologically similar sensilla without any sign of concavity. Shrinkage and deflation happen occasionally during the dehydration process for scanning electron microscopy, which may explain this inconsistency^38^,^39^.

### Sensory pit

The sensory pit is a cave-like depression in the funicular surface that contains several sensilla of the same type (Figs 3E, 7C, 9A, 10B, 11C, 12, 13, 14, 15, 16 and 17). All *Gasterophilus* spp. have the funiculus equipped with numerous sensory pits.

The concept of sensory (or ‘olfactory’) pits needs clarification, as any depression on the cuticle in connection with sensilla are sometimes referred to as a sensory pit^37^,^40^. McAlpine^19^ differentiated between “simple pits” as opposed to the “deep, sac-like invagination of the cuticle of segment 3 containing several trichoid sensilla and opening to the exterior by a relatively small pore”, which Lowne^41^ termed the ‘sacculus’. Similarly, Zhang *et al*.^14^ separated the ‘sensory pit’ as a single-chambered invagination containing a cluster of sensilla from the ‘sacculus’, which was defined as a multi-chambered invagination stretching into the cavity of the antennal funiculus, and often with a complement of different types of sensilla. We propose that the term ‘sensory pit’ should be used to describe a cluster of sensilla located inside a saucer- or bowl-like depression of the cuticular surface with at most one third of their length emerging above the surrounding surface. A fringe of microtrichia is usually found around the edge of a sensory pit. Pezzi *et al*.^42^ used the term ‘olfactory pit’, but as antennal sensilla may have other functions, e.g., hygro- or thermoreception^43^, we prefer the broader term ‘sensory pit’.

### Phylogeny

**Figure 18:**
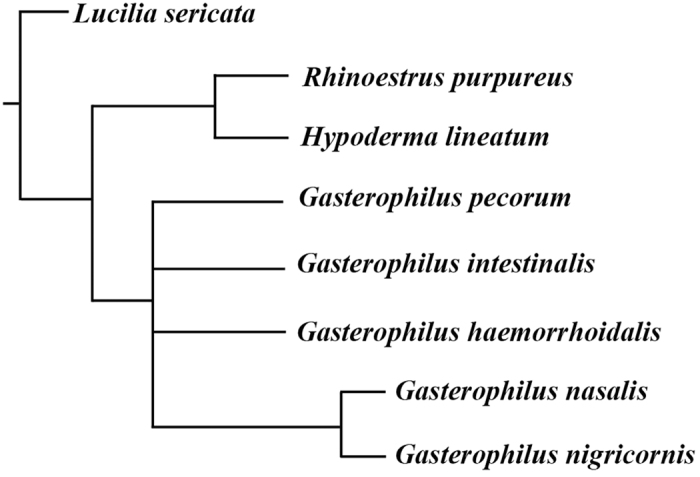
Consensus tree from 2 most parsimonious trees obtained using implicit enumeration, with characters treated as non-additive and equal weighting.

**Figure 19:**
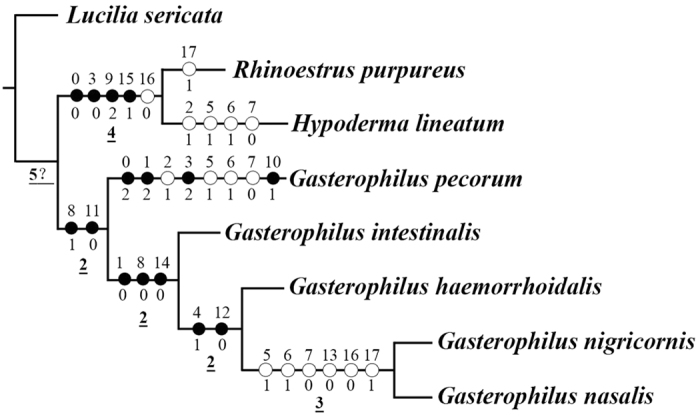
Cladogram obtained using implicit enumeration, with characters treated as non-additive and under implied weighting (k ≥ 1); or with characters treated as additive and under both equal weighting and implied weighting (k ≥ 1) (length = 33 steps; consistency index CI = 69; retention index RI = 72). Numbers refer to characters (above branches) and states (below branches) as discussed in the text and in Appendix 1, black circles represent unique character state changes, white circles represent homoplasious character state changes. Underlined numbers are Bremer support values obtained with characters treated as additive using the implicit enumeration analysis.

Analysing the matrix under equal weights and with all characters treated as non-additive (i.e., unordered) yielded two most parsimonious trees. The strict consensus of the two trees gave a sister-group relationship between the clade Hypodermatinae + Oestrinae and a largely unresolved *Gasterophilus* (Fig. 18). Analysing the data as non-additive under implied weights resulted in a fully resolved tree for all values of k ≥1, which differs from the equally weighted analysis in the topology of *Gasterophilus*, with *G. pecorum* as sister taxon to all other species of *Gasterophilus* (Fig. 19). An identical tree is obtained in analyses with the multistate characters treated as additive, both in the equally weighted analysis and in analyses with implied weighting and for all k-values (i.e., k ≥1). Bremer supports for every node of the minimum-length cladogram are given in Fig. 19.

The genus *Gasterophilus* is supported as a monophyletic group (Fig. 19) based on two synapomorphies: both flat and hair-like microtrichia on antennal funiculus (C8: 1) and the arista with coeloconic sensilla III (C11: 0) (Figs 8C,D,F,G and 9B,C,E,F). A great diversity of antennal sensilla types and locations are evident within this small genus, compared with 3–6 types of antennal sensilla arranged mostly on the funicular surface in other flies^8^,^14^,^15^,^43^,^44^,^45^,^46^. All species of *Gasterophilus* have seven types of sensilla, except for *G. pecorum* having only six types. These cover nearly all types of sensilla (e.g., plaques are not detected) described in cyclorrhaphan flies. Some sensilla can be divided into several subtypes, making a strict calculation of the total number of sensillar types in this genus potentially misleading. Besides, large numbers of sensilla are located in numerous sensory pits and so are more difficult to study. There is increasing evidence that specific types of antennal sensilla have specific functions^47^,^48^. The diverse sensilla and large number of sensory pits in *Gasterophilus* spp., may increase sensitivity to specific odours, while simultaneously maximizing protection of the fragile sensilla from damage^8^,^9^,^10^,^13^,^15^,^49^.

Within *Gasterophilus*, *G. pecorum* branches off at the base, emerging with four autapomorphies: the microtrichia are extensive on the outer side but absent on the inner side of the pedicel (C0: 2) (Fig. 1C), length-width ratio of antennal pedicel more than 0.8 (C1: 2), two antennal pedicel buttons (C3: 2) (13A), and two subtypes of coeloconic sensilla on funiculus (C10: 1) (Fig. 8A,B), and with four homoplasious character states: the pedicel partly enveloping the funiculus (C2: 1), the slim mechanoreceptor (C7: 0) with twisting grooves (C6: 1) but no socket (C5: 1) (Fig. 1D).

*Gasterophilus pecorum* has one more subtype of Co than all the other species included in the present analysis (C10: 1). Co are sensitive to many olfactory cues^48^,^50^,^51^,^52^,^53^,^54^ instead of being only hygro- or thermo-sensitive as speculated previously^55^,^56^,^57^. Further behavioural and electrophysiological studies are required to understand why *G. pecorum* has acquired more types of antennal coeloconic sensilla than other bot flies, but it is noteworthy that *G. pecorum* will deposit eggs at a distance from its host rather than directly on the host^23^, which is probably a derived behaviour that may require additional sensory input to monitor host location.

The second clade in *Gasterophilus* is supported by three synapomorphies: length-width ratio of antennal pedicel less than 0.5 (C1: 0), only flat and grooved microtrichia on antennal funiculus (C8: 0), the presence of auriculate sensilla on antennal funiculus (C14: 0) (Fig. 11A–C). The third clade is supported by two synapomorphies: the pedicel button with distinctly ridgy ring (C4: 1) and coeloconic sensilla III distributed singly on the arista (C12: 0). In this clade, a sister-group relationship of *G. nigricornis* and *G. nasalis* is supported, based on six homoplasious character states: the strong mechanoreceptor (C7: 1) with straight grooves (C6: 0) and socket (C5: 0) (Figs 2E, 3D and 4D), clavate sensillum with a tapering tip (C13: 0), arista with two segments (C16: 0) and distal aristomere with sparse microtrichia (C17: 1).

**Figure 20:**
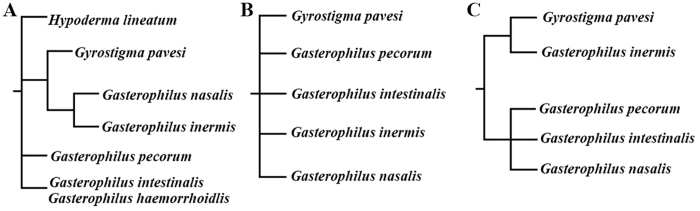
Three rooted cladograms constructed using maximum likelihood analysis of 28S rDNA sequences (**A**), COI sequences (**B**) and 16S rRNA sequences (**C**) (modified from Otranto *et al*.^58^).

No prior study has specifically investigated a species-level phylogeny for *Gasterophilus*. Otranto *et al*.^58^ analysed partial sequence data from the mitochondrial COI gene and the ribosomal genes 16S and 28S in a study of the “differentiation and phylogenesis” of five species of *Gasterophilus*. Unrooted Maximum Likelihood “phylograms” were given based on each gene, but no explicit phylogeny was presented. Rooting their three phylograms (Fig. 20) will generate phylogenies strongly conflicting with the present study (Fig. 19), which may be caused by the sparcity of molecular data. Otranto *et al*.^58^ also suggested that *G. haemorrhoidalis* and *G. intestinalis* could be morphotypes of the same species based on high genetic similarity, in particular of the 28S gene (Fig. 20A). They even considered the main site of development of the third instar larva as sufficiently similar to support conspecificity in spite of known differences^31^. This view did not consider significant evidence that *G. haemorrhoidalis* and *G. intestinalis* are biologically and morphologically quite distinct in several features like oviposition site, oviposition behaviour, details of first instar larva (especially the cephaloskeleton) and adult male and female morphology (for an exquisite treatment see Grunin^31^, which incidentally was not cited by Otranto *et al*.^58^). In the present study, we found significant differences in the antennal morphology of these two species (Figs 14 and 15), such as a distinctly ridgy ring of the pedicellar button and singly distributed aristal Co III in *G. haemorrhoidalis*, while *G. intestinalis* has the alternative state for both these characters (see the results and list of characters in Appendix 1). Actually, the existing similarities between the two species may relate entirely to their shared ancestry, i.e., be symplesiomorphic, because *G. intestinalis* is cladistically subordinate to *G. haemorrhoidalis*, with the latter being more closely related to *G. nigricornis* and *G. nasalis*.

## Additional Information

**How to cite this article**: Zhang, D. *et al*. The antenna of horse stomach bot flies: morphology and phylogenetic implications (Oestridae, Gasterophilinae: *Gasterophilus* Leach). *Sci. Rep.* **6**, 34409; doi: 10.1038/srep34409 (2016).

## Supplementary Material

Supplementary Information

## Figures and Tables

**Figure 17:**
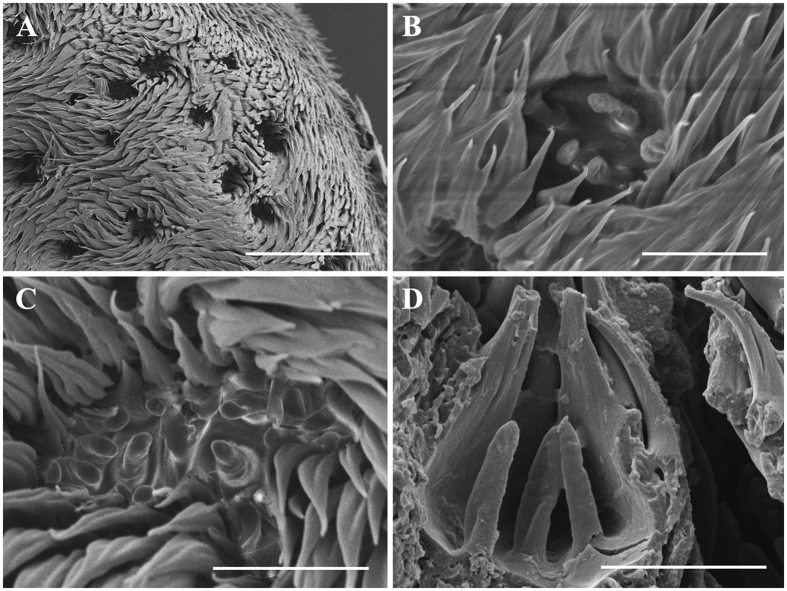
Scanning electron micrographs of sensory pits. (**A**) Overview of sensory pits on funiculus in *Gasterophilus haemorrhoidalis.* (**B**) Dorsal view of a sensory pit with a fringe of microtrichiae in *G. pecorum*. (**C**) Dorsal view of a sensory pit with a fringe of microtrichiae in *G. nasalis*. (**D**) Lateral view of a sensory pit with broken microtrichiae. Scale bars: A = 50 μm, B–D = 10 μm.

**Table 1:** Morphometric data of antennal sensilla in *Gasterophilus haemorrhoidalis* (Linnaeus) (mean ± SD).

Sensilla Type	Sex	Length (μm)	Basal Diameter (μm)	Tip Diameter (μm)
Tr	M	8.06 ± 1.22	1.74 ± 0.15	—
	F	9.59 ± 1.34	1.92 ± 1.22	—
Ba I	M	7.62 ± 0.82	1.96 ± 0.59	—
	F	8.01 ± 1.37	1.99 ± 0.63	—
Ba II	M	2.93 ± 0.46	1.09 ± 0.77	—
	F	3.37 ± 1.09	1.43 ± 0.24	—
Co I	M	4.95 ± 0.67	1.83 ± 0.16	—
	F	5.45 ± 0.52	1.67 ± 0.28	—
Co III	M	6.37 ± 0.54	1.37 ± 0.36	—
	F	6.01 ± 0.97	1.92 ± 0.33	—
Cl	M	8.71 ± 0.32	1.34 ± 0.27	2.01 ± 0.21
	F	12.76 ± 0.90	1.92 ± 0.16	2.90 ± 0.19
Au	M	6.72 ± 0.67	2.37 ± 0.30	—
	F	7.57 ± 0.32	2.69 ± 0.16	—

Au = auriculate sensilla; Ba I = basiconic sensilla I; Ba II = basiconic sensilla II; Cl = clavate sensilla; Co I = coeloconic sensilla I; Co III = coeloconic sensilla III; Tr = trichoid sensilla; F = female; M = male; −= undetermined.

**Table 2:** Morphometric data of antennal sensilla in *Gasterophilus intestinalis* (De Geer) (mean ± SD).

Sensilla Type	Sex	Length (μm)	Basal Diameter (μm)	Tip Diameter (μm)
Tr	M	10.86 ± 1.07	2.01 ± 0.96	—
	F	13.97 ± 0.99	2.23 ± 0.75	—
Ba I	M	7.06 ± 1.43	2.18 ± 0.31	—
	F	8.19 ± 0.95	2.32 ± 0.35	—
Ba II	M	6.80 ± 1.91	1.67 ± 0.19	—
	F	7.44 ± 1.64	1.65 ± 0.24	—
Co I	M	4.51 ± 0.44	2.00 ± 0.09	—
	F	4.82 ± 0.98	1.99 ± 0.95	—
Co III	M	4.02 ± 0.54	1.73 ± 0.19	—
	F	4.19 ± 0.52	1.67 ± 0.28	—
Cl	M	12.20 ± 0.02	1.72 ± 0.31	2.16 ± 0.27
	F	12.31 ± 0.93	1.56 ± 0.14	2.02 ± 0.14
Au	M	4.75 ± 1.16	1.98 ± 0.36	—
	F	5.87 ± 0.79	2.56 ± 0.66	—

Au = auriculate sensilla; Ba I = basiconic sensilla I; Ba II = basiconic sensilla II; Cl = clavate sensilla; Co I = coeloconic sensilla I; Co III = coeloconic sensilla III; Tr = trichoid sensilla I; F = female; M = male; −= undetermined.

**Table 3:** Morphometric data of antennal sensilla in *Gasterophilus nasalis* (Linnaeus) (mean ± SD).

Sensilla Type	Sex	Length (μm)	Basal Diameter (μm)	Tip Diameter (μm)
Tr	M	13.40 ± 1.64	2.26 ± 0.32	—
	F	16.51 ± 0.93	2.04 ± 0.16	—
Ba I	M	9.33 ± 2.08	2.46 ± 0.23	—
	F	11.34 ± 1.97	3.02 ± 0.38	—
Ba II	M	6.72 ± 1.02	1.65 ± 0.24	—
	F	7.10 ± 0.97	2.05 ± 0.25	—
Co I	M	5.05 ± 0.62	3.07 ± 0.16	—
	F	4.24 ± 0.70	2.35 ± 0.15	—
Co III	M	2.66 ± 0.33	2.14 ± 0.21	—
	F	2.58 ± 0.29	2.11 ± 0.19	—
Cl	M	12.30 ± 2.21	2.86 ± 1.02	3.41 ± 1.03
	F	12.16 ± 2.17	3.10 ± 0.96	3.30 ± 1.02
Au	M	5.67 ± 1.05	2.01 ± 0.76	—
	F	7.75 ± 0.75	2.78 ± 0.14	—

Au = auriculate sensilla; Ba I = basiconic sensilla I; Ba II = basiconic sensilla II; Cl = clavate sensilla; Co I = coeloconic sensilla I; Co III = coeloconic sensilla III; Tr = trichoid sensilla I; F = female; M = male; −= undetermined.

**Table 4:** Morphometric data of antennal sensilla in *Gasterophilus pecorum* (Fabricius) (mean ± SD).

Sensilla Type	Sex	Length (μm)	Basal Diameter (μm)	Tip Diameter (μm)
Tr	M	18.47 ± 1.41	2.23 ± 0.16	—
	F	21.23 ± 1.79	2.56 ± 0.34	—
Ba I	M	10.76 ± 2.11	2.16 ± 0.15	—
	F	10.82 ± 1.63	2.21 ± 0.22	—
Ba II	M	6.97 ± 0.78	1.64 ± 0.20	—
	F	7.03 ± 0.81	1.76 ± 0.23	—
Co I	M	2.63 ± 0.52	1.65 ± 0.17	—
	F	3.02 ± 0.61	1.67 ± 0.18	—
Co II	M	6.21 ± 0.93	1.66 ± 0.14	—
	F	6.87 ± 0.87	1.78 ± 0.93	—
Co III	M	5.27 ± 0.92	2.51 ± 0.15	—
	F	5.49 ± 2.04	2.49 ± 0.18	—
Cl	M	10.40 ± 0.66	1.60 ± 0.15	1.38 ± 0.10
	F	10.55 ± 0.75	1.62 ± 0.16	1.41 ± 0.11

Ba I = basiconic sensilla I; Ba II = basiconic sensilla II; Cl = clavate sensilla; Co I = coeloconic sensilla I; Co II = coeloconic sensilla II; Co III = coeloconic sensilla III; Tr = trichoid sensilla I; F = female; M = male; −= undetermined.
