# Supplementary material for: The antenna of horse stomach bot flies: morphology and phylogenetic implications (Oestridae, Gasterophilinae: Gasterophilus Leach)
Source: Sci Rep. 2016 Oct 5;6:34409. doi: 10.1038/srep34409 (PMC5050557; doi:10.1038/srep34409)
Supplement: Supplementary Information [file srep34409-s1.pdf]

Title:

The antenna of horse stomach bot flies: morphology and phylogenetic implications (Oestridae, Gasterophilinae: *Gasterophilus* Leach)

Authors:

Dong Zhang, Xinyu Li, Xianhui Liu, Qike Wang, Thomas Pape

Appendix 1

Characters and observations

0. *Antennal pedicel, microtrichosity*: largely absent on both sides (0) / absent on inner side but extensive on the outer side (1) / extensive on both sides (2)

   The pedicel is largely without microtrichia in the Oestrinae and Hypodermatinae[8]. In most calyptrates and in the four species of *Gasterophilus* (except for *G. pecorum*), the pedicel is covered with microtrichia on both sides (Figs. 2D, 3C, 4C); whereas the microtrichia are distributed only on the inner side in *G. pecorum* (Fig. 1C).

1. *Antennal pedicel, length-width ratio*: < 0.5 (0) / 0.5 < x < 0.8 (1) / > 0.8 (2)

   The pedicel is always short in *Gasterophilus* species (Figs. 2 A–B, D, 3A–C, 4A–C), except for *G. pecorum*, which has an elongated pedicel (Fig. 1A–C). It is an unusual character state in Oestridae as well as in other Calyptratae[10,14,15]. In many other calyptrates, the pedicel is relatively long, with a length-width ratio more than 0.5 but usually less than 0.8 as is the case in *Lucilia sericata*.

2. *Antennal pedicel*: not enveloping funiculus (0) / at least partly enveloping funiculus (1)

   The funiculus is partly enveloped by the pedicel in all Hypodermatinae and in *G. pecorum*.

3. *Antennal pedicel, button number*: none (0) / one (1) / two (2)

   The pedicel button is a sensilla found at the base of the pedicellar seam in schizophoran Diptera[15,19]. Two pedicellar buttons are found in *G. pecorum*, one in the remaining species of *Gasterophilus*, while the button appears to be absent in the Hypodermatinae and Oestrinae[9,10].

4. *Pedicel button ring, shape*: slightly convex (0) / distinctly ridgy (1)

   A distinctly ridgy ring of the pedicellar button is seen in *G. haemorrhoidalis*, *G. nasalis* and *G. nigricornis*.

5. *Antennal mechanoreceptor socket*: well-developed (0) / indistinct (1)

   This and the following two characters separate the unique configuration of two kinds of antennal mechanoreceptors in Oestridae. One type is the slim and twisting mechanoreceptors with/without distinct socket and the other is the strong and straight one with a well-developed socket. In other calyptrate species, different configurations can be found[9,10].

6. *Antennal mechanoreceptor grooves, direction*: straight (0) / twisting (1)

7. *Antennal mechanoreceptor, shape*: slim(0) / strong (1)

   Mechanoreceptors are slim in *Hypoderma lineatum*, *G. pecorum*, *G. nigricornis* and *G. nasalis* (Figs. 1C–D, 2C–E), while strong in *Rhinoestrus purpureus*, *G. intestinalis* and *G. haemorrhoidalis* (Figs. 3C–D, 4C–D).

8. *Antennal funiculus microtrichia, shape*: only flat and grooved (0) / both flat and hair-like (1) / only hair-like (2)

   The flat and grooved microtrichia are found in all five species of *Gasterophilus* studied. The hair-like microtrichia, which are shared by the majority of flies, are found in *G. pecorum* and the two outgroup taxa.

9. *Antennal funiculus sensilla, arrangement*: mainly on surface but some clustered in sensory pits (0) / both on surface and sensory pits (1) / mainly in sensory pits but some scattered on the surface (2)

   The condition of antennal funiculus sensilla located mainly on the surface is common in calyptrate species[14]. In *H. lineatum* and *R. purpureus*, the funiculus sensilla are largely clustered in sensory pits but with some scattered on the surface[9] in species of *Gasterophilus*, the funiculus has a large number of sensilla both on surface and sensory pits (Figs. 6–12).

10. *Coeloconic* se*nsilla on funiculus, number of subtypes*: one (0) / two (1)

    Coeloconic sensilla of Calyptratae can always be divided into several subtypes due to their variable morphology[13]. In *Gasterophilus*, *G. pecorum* has one more subtype of coeloconic sensilla than its congeners as well as of the current outgroups.

11. *Coeloconic sensilla III*: present (0) / absent (1)

    Coeloconic sensilla III are found on the antennal arista in all the *Gasterophilus* spp. but have not been documented in any other calyptrate taxa.

12. *Coeloconic sensilla III, distribution on surface*: singly (0) / clustered (1)

    In *G. intestinalis* and *G. pecorum,* coeloconic sensilla III are clustered in shallow depressions, while in *G. haemorrhoidalis*, *G. nasalis*, and *G. nigricornis* coeloconic sensilla III are distributed singly.

13. *Clavate sensillum, shape*: with a short, tapering tip (0) / abruptly tapered and tip broadly rounded (1)

    The clavate sensillum has a tapering tip in *H. lineatum*, *R. purpureus*, *G. nigricornis* and *G. nasalis*; which is not evident in *G. pecorum*, *G. intestinalis* and *G. haemorrhoidalis*.

14. *Auriculate sensilla*: present (0) / absent (1)

    Auriculate sensilla are found in all species of *Gasterophilus* examined except for *G. pecorum.* They have not been found in *H. lineatum* and *R. purpureus*.

15. *Trichoid sensilla, number*: numerous, covering the funiculus (0) / very few (1)

    Trichoid sensilla are always the most numerous sensilla on the funicular surface[14], but in *H. lineatum* and *R. purpureus*, the trichoid sensilla are found in very low numbers[9].

16. *Aristal segments, number*: two (0) / three (1)

    The arista usually has three segments in cyclorrhaphan flies. In *H. lineatum, R. purpureus*, *G. nigricornis* and *G. nasalis*, the arista has only two segments, a short basal segment and a long distal segment.

17. *Aristal distal segment, number of microtrichia*: present, sparse (0) / absent (1)

    The aristal distal segment possesses long or short microtrichia in many calyptrate species. In oestrid flies, the aristal microtrichia are always sparse. In *R. purpureus*, *G. nigricornis* and *G. nasalis*, the aristal distal segment is entirely devoid of microtrichia.

Appendix 2

Morphological data matrix of antennal structures in *Gasterophilus* species. The coding “-” is used for both unknown and inapplicable states.

**Species**	0										1							
	0	1	2	3	4	5	6	7	8	9	0	1	2	3	4	5	6	7
Lucilia sericata	2	1	0	1	0	0	0	1	2	0	0	1	-	-	1	0	1	0
Rhinoestrus purpureus	0	1	0	0	-	0	0	1	2	2	0	1	-	0	1	1	0	1
Hypoderma lineatum	0	1	1	0	-	1	1	0	2	2	0	1	-	0	1	1	0	0
Gasterophilus pecorum	1	2	1	2	0	1	1	0	1	1	1	0	1	1	1	0	1	0
Gasterophilus intestinalis	2	0	0	1	0	0	0	1	0	1	0	0	1	1	0	0	1	0
Gasterophilus haemorrhoidalis	2	0	0	1	1	0	0	1	0	1	0	0	0	1	0	0	1	0
Gasterophilus nigricornis	2	0	0	1	1	1	1	0	0	1	0	0	0	0	0	0	0	1
Gasterophilus nasalis	2	0	0	1	1	1	1	0	0	1	0	0	0	0	0	0	0	1
